# Function of the PHA-4/FOXA transcription factor during *C. elegans *post-embryonic development

**DOI:** 10.1186/1471-213X-8-26

**Published:** 2008-02-29

**Authors:** Di Chen, Donald L Riddle

**Affiliations:** 1 Division of Biological Sciences, University of Missouri, Columbia, MO 65211, USA; 2 Michael Smith Laboratories, University of British Columbia, Vancouver, BC V6T 1Z4, Canada; 3 Buck Institute for Age Research, 8001 Redwood Blvd, Novato, CA 94945, USA

## Abstract

**Background:**

*pha-4 *encodes a forkhead box (FOX) A transcription factor serving as the *C. elegans *pharynx organ identity factor during embryogenesis. Using Serial Analysis of Gene Expression (SAGE), comparison of gene expression profiles between growing stages animals and long-lived, developmentally diapaused dauer larvae revealed that *pha-4 *transcription is increased in the dauer stage.

**Results:**

Knocking down *pha-4 *expression by RNAi during post-embryonic development showed that PHA-4 is essential for dauer recovery, gonad and vulva development. *daf-16*, which encodes a FOXO transcription factor regulated by insulin/IGF-1 signaling, shows overlapping expression patterns and a loss-of-function post-embryonic phenotype similar to that of *pha-4 *during dauer recovery. *pha-4 *RNAi and *daf-16 *mutations have additive effects on dauer recovery, suggesting these two regulators may function in parallel pathways. Gene expression studies using RT-PCR and GFP reporters showed that *pha-4 *transcription is elevated under starvation, and a conserved forkhead transcription factor binding site in the second intron of *pha-4 *is important for the neuronal expression. The vulval transcription of *lag-2*, which encodes a ligand for the LIN-12/Notch lateral signaling pathway, is inhibited by *pha-4 *RNAi, indicating that LAG-2 functions downstream of PHA-4 in vulva development.

**Conclusion:**

Analysis of PHA-4 during post-embryonic development revealed previously unsuspected functions for this important transcriptional regulator in dauer recovery, and may help explain the network of transcriptional control integrating organogenesis with the decision between growth and developmental arrest at the dauer entry and exit stages.

## Background

At the second larval molt, *C. elegans *may arrest development at the dauer stage in response to starvation and overcrowding, but can resume development to the adult when an environment favoring growth is encountered [1]. Entry into and exit from the dauer stage are determined by environmental cues, such as temperature, food supply and a constitutively released, dauer-inducing pheromone [2-4]. Genes involved in dauer formation are called *daf *genes. Dauer-constitutive (Daf-c) mutants form dauer larvae in an environment with abundant food, whereas dauer-defective (Daf-d) mutants fail to enter the dauer stage when they are starved or overcrowded. Most Daf-c mutants are temperature-sensitive, revealing the natural temperature dependence of dauer formation [5].

Many *daf *genes have been ordered in a branched genetic pathway based on genetic epistasis [5]. Three functionally overlapping pathways, including TGF-β, insulin/IGF-1, and cyclic GMP signaling pathways, are involved in responding to environmental cues [6]. DAF-7, a member of the TGF-β superfamily of protein growth factors, signals through downstream receptor kinases, SMAD transcription factors and the DAF-12 nuclear hormone receptor to inhibit dauer formation and promote reproductive development [7-12]. The *daf-2 *gene encodes an IGF-1 receptor, which functions through downstream kinases to phosphorylate the DAF-16/FOXO transcription factor [13-17], and to influence the biosynthesis of ligands for DAF-12 [18-21].

As a step toward understanding the genetic basis of diapause and longevity, Jones *et al*. [22] compared gene expression profiles of dauer larvae and mixed-stage, growing populations by SAGE. Transcripts that were enriched either in dauer larvae or in mixed stages were identified [22]. SAGE tags corresponding to *pha-4*, which encodes a FOXA transcription factor homolog, were detected in the dauer stage (8 tags), but not in mixed stages (no tags).

PHA-4 is regarded as the organ identity factor for the *C. elegans *pharynx [23-25]. In *pha-4 *mutants, pharyngeal cells are transformed into ectoderm and the development of mutant animals is arrested after hatching [25]. *pha-4 *mRNA is highly enriched in both the pharyngeal and intestinal primordia of the embryo, and low levels of *pha-4 *transcripts can be detected in the L3/L4 larval somatic gonad [26]. Candidate PHA-4 target genes in pharyngeal development have been identified [27], and analysis of target promoter sequences revealed several *cis*-regulatory elements. These elements are targets of unknown transcription factors, which function coordinately with PHA-4 to modulate gene expression in different pharyngeal cell types and at different developmental stages [28,29]. Ao *et al*. [28] found that the DAF-12 nuclear hormone receptor is one of these transcription factors. DAF-12 and PHA-4 function together to either activate or inhibit *myo-2*, the pharyngeal muscle gene, in response to environmental and developmental cues [28]. Thus, PHA-4 is an important regulator that is expressed in several cell types and controls a wide range of gene expression.

To assess PHA-4 functions in dauer larvae, we treated Daf-c mutants with *pha-4 *RNAi. We found that PHA-4 is essential for dauer recovery, gonad and vulva development. The hermaphrodite somatic gonad includes distal tip cells, gonadal sheath cells, spermathecae and uterus. Distal tip cells regulate gonadal arm elongation and the switch from mitosis to meiosis in germline cells through the GLP-1/Notch signaling pathway [30]. Gonadal sheath cells have physical contacts with the germline; the muscle components convey contractile properties required for ovulation. Moreover, the sheath cells, spermathecal and uterine precursor cells play regulatory roles in germline development, such as promoting germline proliferation, exit from pachytene, and/or gametogenesis [31]. Identification of PHA-4 targets in the somatic gonad should help explain the PHA-4 gonadal phenotype. The vulval transcription of *lag-2*, which encodes a ligand for the LIN-12/Notch lateral signaling pathway, is inhibited by *pha-4 *RNAi, indicating that LAG-2 functions downstream of PHA-4 in vulva development.

## Results

### PHA-4 is required for dauer recovery, gonad and vulva development

**Figure 1:**
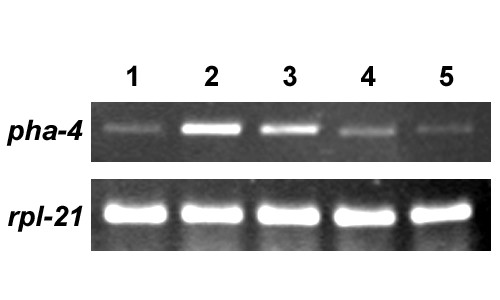
***pha-4 *transcripts are enriched in dauer larvae**. Semi-quantitative RT-PCR experiments were performed to determine levels of *pha-4 *and *rpl-21 *mRNA. *rpl-21*, which encodes a ribosomal protein, serves as the internal control for equal loading. Experiments were performed three times with consistent results using three independent RNA preparations. Lane 1, mixed stages; lane 2, dauer; lane 3, 4 and 5, 2 hours, 24 hours and 36 hours after placement in food, respectively. Dauer larvae begin to feed approximately three hours after exposure to food.

SAGE revealed that *pha-4 *transcription is elevated in dauer larvae [22], suggesting that, in addition to pharyngeal organogenesis, PHA-4 may function in dauer formation or recovery. We used semi-quantitative RT-PCR to compare *pha-4 *transcript levels in mixed-stages, dauer, and at different times during dauer recovery. *pha-4 *transcripts were much more abundant in the dauer stage than in mixed stages, and were also detected shortly after dauer larvae started to recover. *pha-4 *transcripts decreased during resumption of development (Fig. 1).

**Figure 2:**
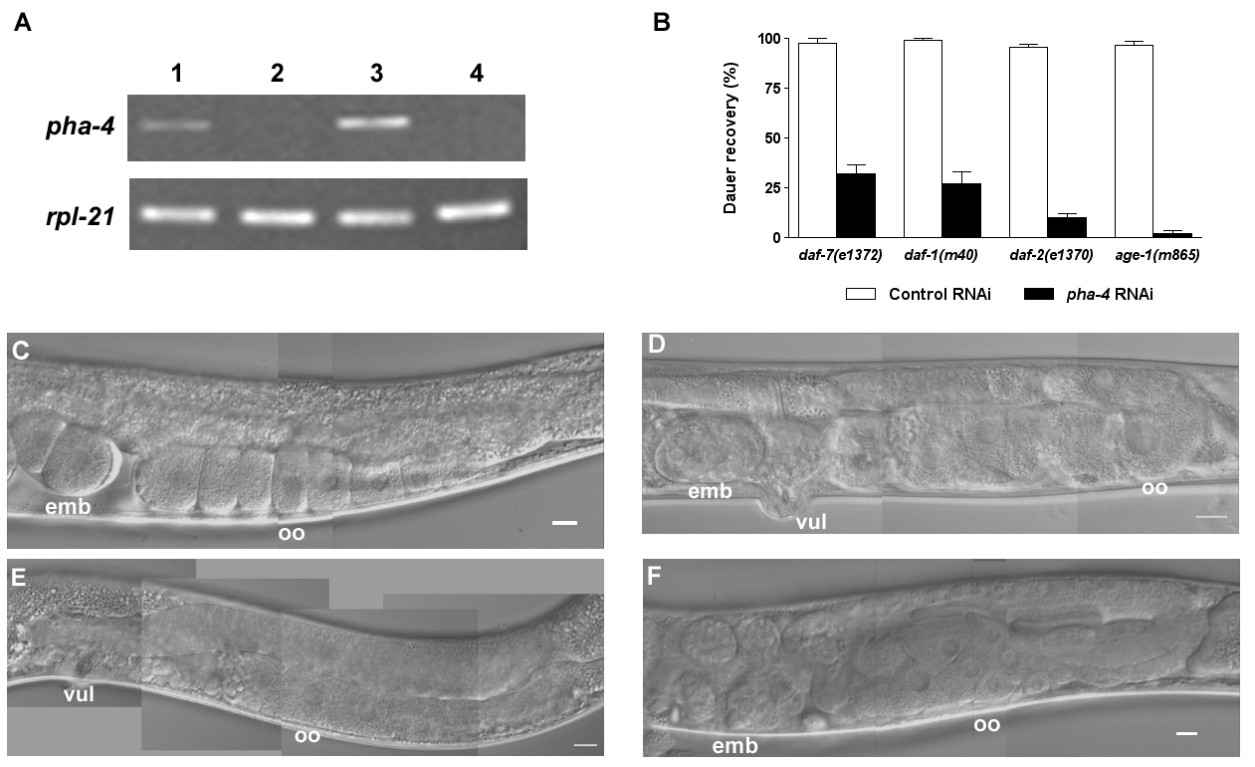
**PHA-4 is required for dauer recovery, gonad and vulva development**. **(A) ***pha-4 *RNAi treatment efficiently inhibits *pha-4 *transcription. Semi-quantitative RT-PCR shows *pha-4 *and *rpl-21 *transcription levels in *daf-7(e1372) *and *daf-2(e1370) *dauer larvae treated with the RNAi control (lanes 1, 3) and *pha-4 *RNAi (lanes 2, 4). RT-PCR experiments were performed three times with consistent results using three independent RNA preparations. **(B) **PHA-4 is needed for dauer recovery. Dauer larvae formed constitutively at 25°C in the presence of *pha-4 *RNAi from hatching showed significantly decreased recovery upon downshift to 15°C compared to control *daf-7(e1372)*, *daf-1(m40)*, *daf-2(e1370)*, and *age-1(m865) *mutants. Percentages of dauer recovery, numbers of animals scored, and *p *values for *t*-tests were shown in Table 1. The entire experiment was performed twice with triplicates for each treatment. **(C-F) ***pha-4 *is involved in gonad and vulva development. **(C) **Control post-dauer *daf-7(e1372) *adult; **(D) ***pha-4 *RNAi-treated post-dauer *daf-7(e1372) *adult; **(E) **control post-dauer *daf-2(e1370) *adult; and **(F) ***pha-4 *RNAi-treated post-dauer *daf-2(e1370) *adult. The Daf-c mutants all exhibit similar gonad and vulva defects with variable expressivity. Anterior is left and dorsal is up. dtc, distal tip cell; germ, germline; oo, oocytes; emb, embryos; vul, vulva. Scale bars, 10 μm.

To determine PHA-4 function in the dauer stage, we knocked-down *pha-4 *expression by feeding Daf-c mutant animals *E. coli *expressing *pha-4 *double-stranded RNA [32] from the time of hatching from the egg. As a control, we fed the same strains with *E. coli *that carries the RNAi vector (L4440) without any insert. Semi-quantitative RT-PCR showed that *pha-4 *transcripts were efficiently reduced by the RNAi treatment (Fig. 2A).

Dauer larvae with *pha-4 *RNAi treatment showed normal dauer characteristics, including SDS resistance and a constricted pharynx (not shown), suggesting that PHA-4 is not required for dauer formation. However, when the dauer larvae were transferred to an environment favoring growth (fresh food and low temperature), they exhibited decreased recovery compared to the control, 2 – 32% vs. 96 – 99% (Fig. 2B, Table 1). *pha-4 *RNAi-treated animals that resumed development to the adult had abnormal oocytes and embryos, and protruding vulvae (Fig. 2D, 2F). Adults fed the RNAi control under the same conditions were normal (Fig. 2C, 2E). Thus, PHA-4 plays essential roles in dauer recovery and gonad/vulva development.

**Table 1:** *pha-4 *and *daf-16 *are required for dauer recovery

**Genotype**	**RNAi**	**Dauer recovery (%) ^a^**	**n ^b^**	***p***
***pha-4 *is required for dauer recovery**				
*daf-7(e1372)*	control	97.9 ± 2.1	71	/
*daf-7(e1372)*	*pha-4*	32.1 ± 4.4	76	0.0002 ^c^
*daf-1(m40)*	control	99.3 ± 0.7	89	/
*daf-1(m40)*	*pha-4*	27.4 ± 6.0	89	0.0003 ^c^
*daf-2(e1370)*	control	96.0 ± 1.4	108	/
*daf-2(e1370)*	*pha-4*	10.4 ± 1.9	87	<0.0001 ^c^
*age-1(m865)*	control	96.6 ± 1.9	86	/
*age-1(m865)*	*pha-4*	2.3 ± 1.2	84	<0.0001 ^c^
				
***daf-16 *is required for dauer recovery**				
*daf-7(e1372)*	/	97.3 ± 1.3	78	/
*daf-16(mgDf47); daf-7(e1372)*	/	41.0 ± 3.6	92	0.0001 ^d^
				
**PHA-4 and DAF-16 function in parallel during dauer recovery**				
*daf-7(e1372)*	control	97.3 ± 1.5	111	/
*daf-7(e1372)*	*pha-4*	44.0 ± 2.4	107	<0.0001 ^c^
*daf-16(mgDf47); daf-7(e1372)*	control	53.5 ± 6.2	109	0.0023 ^e^
*daf-16(mgDf47); daf-7(e1372)*	*pha-4*	9.1 ± 2.1	101	0.0024 ^c^ 0.0004 ^f^

^a ^Mean percentages of dauer recovery ± standard errors.

^b ^Number of animals scored.

^c ^Daf-c mutants treated with control RNAi vs. *pha-4 *RNAi; *p *values for *t*-tests were calculated using the GraphPad Prism 4 software..

^d ^*daf-7 *vs. *daf-16; daf-7 *treated with OP50 *E. coli *food.

^e ^*daf-7 *vs. *daf-16, daf-7 *treated with control RNAi.

^f ^*daf-7 *vs. *daf-16; daf-7 *with *pha-4 *RNAi.

**Figure 3:**
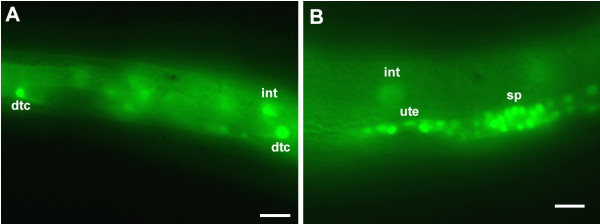
***pha-4 *is expressed in the somatic gonad. (A) ***pha-4::gfp *expression in the distal tip cells and in the intestine of a *daf-2(e1370) *dauer larva. **(B) ***pha-4::gfp *expression in the uterus and spermathecae of a *daf-2(e1370) *L4 larva. Anterior is left and dorsal is up. dtc, distal tip cell; sp, spermatheca; ut, uterus; int, intestine. Scale bars, 10 μm.

To assess the possible role of PHA-4 in gonad and vulva development, a *pha-4::gfp *translational fusion carrying the 1.5 kb promoter (the first two exons and introns, and part of the third exon of *pha-4 *fused in-frame with the *gfp *coding region) was injected into the germline of *daf-2(e1370)*. GFP was present in distal tip cells (DTCs), intestinal cells (Fig. 3A) and neurons (not shown) in dauer larvae, and in the spermathecae and uteri of L4 larvae (Fig. 3B).

### DAF-16 is also important for dauer recovery, gonad and vulva development

**Figure 4:**
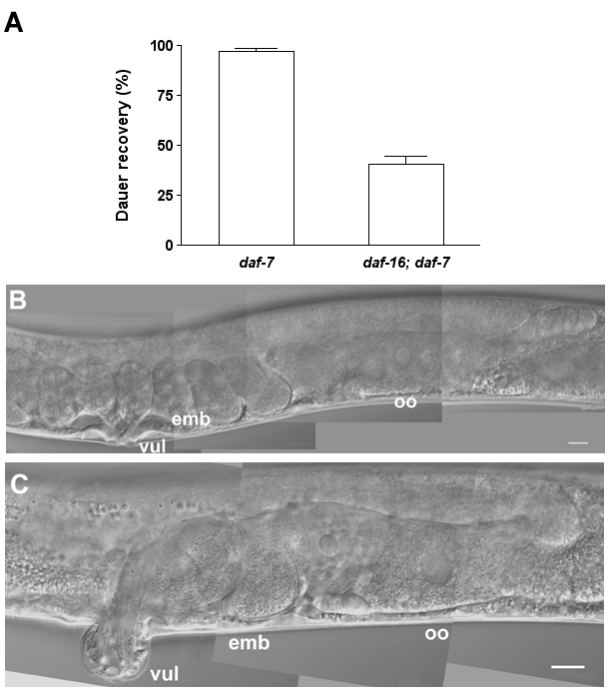
**DAF-16 regulates dauer recovery, gonad and vulva development**. **(A) **DAF-16 is involved in dauer recovery. The *daf-16(mgDf47); daf-7(e1372) *double mutant has significantly decreased dauer recovery percentage compared to *daf-7(1372)*. Percentages of dauer recovery, numbers of animals scored, and *p *values for *t*-tests are shown in Table 1. The entire experiment was performed twice with triplicates for each treatment. **(B-C) **DAF-16 is involved in post-dauer gonad and vulva development. **(B) **A *daf-7(e1372) *post-dauer adult, and **(C) **a *daf-16(mgDf47); daf-7(e1372) *post-dauer adult. Anterior is left and dorsal is up. vul, vulva; oo, oocytes; and emb, embryos. Scale bars, 10 μm.

Previous studies showed that DAF-16B, one of the *daf-16 *gene products, is expressed in the pharynx, somatic gonad and tail neurons [33]. The overlapping expression patterns of *daf-16b *and *pha-4 *and the essential roles of DAF-16 during dauer development suggest the possibility that DAF-16 may be involved in dauer recovery. Since *daf-16 *is a Daf-d mutant, we used a *daf-16(mgDf47); daf-7(e1372) *strain to examine dauer recovery and adults recovered from the dauer stage. The *daf-16(mgDf47) *null mutant carries the *daf-16 *mutation lacking the coding sequence for DNA binding domains [33]. The *daf-7 *Daf-c mutant affects the TGF-β pathway, and is not suppressed by *daf-16 *mutations [34]. The *daf-16; daf-7 *dauer larvae formed constitutively at 25°C showed significantly decreased recovery upon downshift to 15°C compared to the *daf-7(e1372) *dauer larvae (Fig. 4A, Table 1). Animals, which resumed development to the adult, showed defects in the gonad and vulva similar to *pha-4 *RNAi-treated animals (Fig. 4B, 4C).

### DAF-16 and PHA-4 function in parallel pathways to regulate dauer recovery

Since *pha-4 *transcripts are elevated in dauer larvae, transcription factors in dauer signaling pathways are candidates for promoting *pha-4 *expression in dauer larvae. The overlapping expression patterns and similar loss-of-function phenotypes of *daf-16 *and *pha-4 *suggest the possibility that DAF-16 may be a regulator of *pha-4 *transcription.

**Figure 5:**
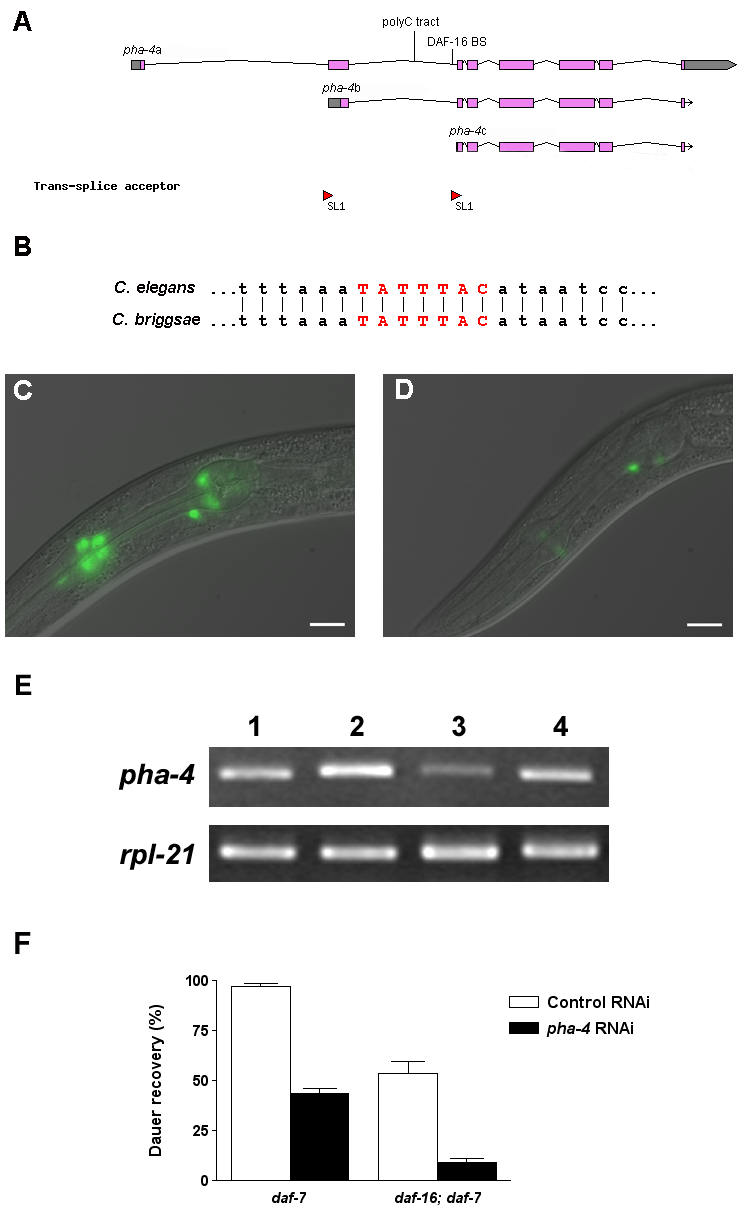
**DAF-16 and PHA-4 function in parallel during dauer recovery**. **(A-E) **Transcriptional regulation of *pha-4 *expression. **(A) ***pha-4 *gene structure (adapted from [49]). **(B) **The potential DAF-16 binding site in the second intron of *pha-4 *and flanking sequences are conserved in the corresponding intron of *C. briggsae*. The potential DAF-16 binding site is shown in red, capital letters. **(C) **A *daf-2(e1370) *L4 larva carrying a *pha-4::gfp *reporter with the potential DAF-16 binding site shows *gfp *expression in head neurons. **(D) **A *daf-2(e1370) *L4 larva carrying a *pha-4::gfp *reporter with the potential DAF-16 binding site mutated shows diminished *gfp *expression in head neurons. Two transgenic lines were made from each construct. 20 animals were examined from each transgenic line. Over 50% of animals showed representative expression patterns as shown in this panel. Pictures were taken using the same exposure time. **(E) **Increased *pha-4 *transcription upon starvation. Semi-quantitative RT-PCR experiments were performed to assess *pha-4 *transcript levels in well-fed N2 L3 larvae with low DAF-16 activity (lane 1); starved N2 L3 larvae with high DAF-16 activity (lane 2); well-fed *daf-16(mgDf47) *L3 larvae and starved *daf-16(mgDf47) *L3 larvae with no DAF-16 activity (lanes 3 and 4). RT-PCR experiments were performed three times with consistent results using three independent RNA preparations. **(F) **The *daf-16 *mutation and *pha-4 *RNAi have additive effects on dauer recovery. A *daf-16(mgDf47); daf-7(e1372) *double mutant treated with *pha-4 *RNAi showed more severe dauer recovery defects compared to either the *daf-16(mgDf47); daf-7(e1372) *mutant control or the *daf-7(e1372) *mutant treated with *pha-4 *RNAi. Percentages of dauer recovery, numbers of animals scored, and *p *values for *t*-tests are shown in Table 1. The entire experiment was performed twice with triplicates for each treatment.

*pha-4 *is transcribed into three major transcripts: *pha-4*a, b and c [26]. *pha-4 *b and c are *trans*-spliced with the SL1 leader mRNA at the beginning of exons 2 and 3, respectively (Fig. 5A). Introns 1 and 2 are large (2247 bp and 1329 bp, respectively), considering that the median size of confirmed introns in the *C. elegans *genome is 65 bp [35]. These introns may contain promoters for *pha-4*b and *pha-4*c, or may carry *cis*-regulatory elements. The second intron contains a potential DAF-16 binding site localized near the 5' -end of the *pha-4*c coding region (Fig. 5A). This site and flanking sequences are conserved in the corresponding intron of the *pha-4 *homolog in *C. briggsae *(Fig. 5B).

We mutated the element in the *pha-4::gfp *construct, but did not observe altered *gfp *expression in transgenic animals (data not shown). It is possible that *pha-4a *expression is not affected by the mutation. To test whether *pha-4*b and *pha-4*c are regulated through this binding site, we cloned a 3.8-kb DNA fragment of *pha-4 *(which carries the first two introns, the second exon and part of the third exon) in frame with the *gfp *coding sequence. We then mutated the potential DAF-16 binding site, and injected these two constructs into *daf-2 *animals. Animals carrying the construct with the normal DAF-16 binding site showed *gfp *expression only in head neurons (Fig. 5C); whereas animals carrying the mutated DAF-16 binding site showed very weak *gfp *expression in the same cells (Fig. 5D). This suggests that the potential DAF-16 binding site in the second intron of *pha-4 *has regulatory activity *in vivo*.

DAF-16 is activated and localized to the nucleus shortly after food deprivation [36]. We compared *pha-4 *mRNA levels between well-fed and starved L3 larvae, the alternative third stage to dauer larvae. Starved wild-type L3 larvae, which have high DAF-16 activity, had higher levels of *pha-4 *transcripts than well-fed wild-type L3 larvae, which have low DAF-16 activity (Fig. 5E). However, starvation also increases *pha-4 *transcription in the *daf-16(mgDf47) *null mutant background, although both well-fed and starved *daf-16 *animals showed slightly lower *pha-4 *transcription compared to wild-type N2 animals (Fig. 5E). These results suggest that there may be more than one transcriptional regulator promoting *pha-4 *transcription in response to starvation.

To test the genetic interaction between DAF-16 and PHA-4, we treated the *daf-16; daf-7 *double mutant, which is Daf-c, with the control or *pha-4 *RNAi in dauer recovery assays. *pha-4 *RNAi treatment further reduced recovery of *daf-16; daf-7 *dauer larvae (Fig. 5F, Table 1). This result suggests that DAF-16 and PHA-4 may function in parallel pathways to regulate dauer recovery.

### Knocking-down *pha-4 *by RNAi inhibits *lag-2 *transcription in vulval cells

*lag-2 *is a candidate PHA-4 target, since *pha-4 *and *lag-2 *are both expressed in DTCs in dauer larvae, and there are two potential PHA-4 binding sites in the 1 kb *lag-2 *promoter. *lag-2 *encodes a ligand for Notch signaling pathways in gonad and vulva development [37,38]. LAG-2 expression in DTCs regulates entry into mitosis versus meiosis through the GLP-1/Notch signaling pathway [38]. Dauer larvae carrying an integrated *lag-2::gfp *reporter exhibited *lag-2 *expression in IL1 neurons and DTCs (data not shown).

We crossed the integrated *lag-2::gfp *reporter into the *daf-2(e1370) *mutant to test the effect of *pha-4 *RNAi on *lag-2 *expression in DTCs and to assess possible post-dauer gonadal defects. We found that *lag-2::gfp *expression in DTCs and IL1 neurons is not affected by *pha-4 *RNAi treatment, nor did *lag-2 *RNAi-treated animals show post-dauer gonad defects that were observed in animals treated with *pha-4 *RNAi (data not shown). GLD-1, a KH motif-containing RNA binding protein, functions downstream of LAG-2 in the GLP-1/Notch signaling pathway in germline development [39]. *pha-4 *RNAi treatment also does not affect GLD-1 expression in the *daf-7(e1372) *mutant (data not shown). Hence, PHA-4 may not function through the LAG-2-GLP-1/Notch signaling in gonad development.

**Figure 6:**
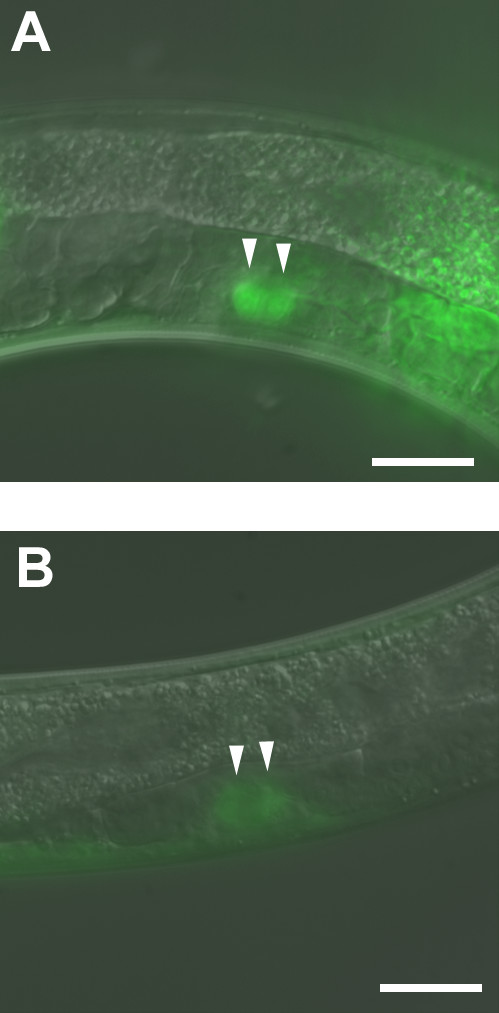
**PHA-4 is required for vulval transcription of *lag-2***. DR2429 *daf-2(e1370); qIs56(unc-119 lag-2::gfp) *animals were treated with control or *pha-4 *RNAi after hatching at 25°C until dauer larvae formed constitutively. The dauer larvae were then transferred to fresh RNAi plates and incubated at 15°C for 2 days until animals resumed development to the late L4 stage. Two out of four P6.p descendant cells with *lag-2::gfp *expression are indicated by arrow heads in a control DR2429 post-dauer L4 larva **(A) **and a DR2429 post-dauer L4 larva with *pha-4 *RNAi treatment **(B)**. The other two P6.p descendant cells show similar *lag-2::gfp *expression patterns. The entire experiment was performed twice with 40 animals examined for each treatment. 37 out of 40 *pha-4 *RNAi-treated DR2429 post-dauer L4 larvae showed obviously diminished *lag-2::gfp *in four P6.p descendant cells compared to control animals. Anterior is left and dorsal is up. Scale bars, 10 μm.

*lag-2 *is also expressed in P6.p and its descendants, the cells that adopt the 1° vulval fate [37,40,41]. LAG-2 is one of the ligands that function through LIN-12/Notch lateral signaling to promote P5.p and P7.p to adopt 2° vulval fate [37]. Previous studies showed that a *lag-2 *temperature-sensitive (ts) mutant has a protruding vulva (Pvl) when it is grown at the restrictive temperature [42]. The Pvl phenotype is similar to that of *pha-4 *RNAi-treated animals. Thus, it is possible that LAG-2 functions downstream of PHA-4 in vulva development. Using the same *lag-2::gfp *transgene as shown in previous studies [40,41], we observed *lag-2::gfp *expression in four P6.p descendant cells in L4 larvae. *pha-4 *RNAi greatly decreased *lag-2::gfp *expression in those P6.p descendant cells (Figure 6). Therefore, PHA-4 is required for *lag-2 *expression in vulval cells *in vivo*. Although electrophoretic mobility shift assays (EMSAs) indicated that PHA-4 binds to the *lag-2 *promoter *in vitro *(data not shown), it is not clear whether PHA-4 directly promotes *lag-2 *transcription in vulval precursor cells. Taken together, the data show that PHA-4 is important for *lag-2 *transcription in vulva development, but does not affect *lag-2 *expression in distal tip cells for germline development.

## Discussion

### PHA-4 plays essential roles in post-dauer development

PHA-4 is expressed in every pharyngeal cell during embryonic development, and it directly regulates expression of many pharyngeal genes [23,24,27-29]. SAGE and RT-PCR data showed that *pha-4 *transcription is elevated in the dauer stage. Our RNAi analysis revealed that PHA-4 plays essential roles in dauer recovery, gonad and vulva development. Thus, PHA-4 serves as a key regulator to control different aspects of embryonic and larval development. *pha-4 *is expressed in the somatic gonad. Previous studies suggested that there is an unknown signal from gonadal sheath cells, uterus and spermathecae precursor cells that regulates germline development [31]. Identification of PHA-4 targets in gonad and vulva development may help characterize this unknown signal.

### DAF-16 and PHA-4 function in parallel pathways during dauer recovery

Transcription factors from dauer pathways are potential regulators of *pha-4 *transcription since *pha-4 *mRNA levels are increased in dauer larvae. We focused on DAF-16 because 1) *daf-16 *and *pha-4 *have overlapping expression patterns, 2) *daf-16 *and *pha-4 *have similar loss-of-function phenotypes, and 3) there is a conserved, potential DAF-16 binding site in the non-coding region of *pha-4*. We have shown that this potential DAF-16 binding site has regulatory function on *pha-4 *expression in head neurons. However, this regulation is unlikely to be important for the RNAi phenotypes we observed since gene expression in wild-type neurons is refractory to RNAi.

*pha-4 *shows increased transcription under starvation, which also causes increased DAF-16 activities. This observation is consistent with the recent study that PHA-4 plays important roles in dietary restriction-mediated life span extension and *pha-4 *transcription is increased upon dietary restriction or in the *eat-2 *mutant, which serves as a genetic mimic of dietary restriction [43]. However, the increased *pha-4 *transcription upon starvation is independent of *daf-16*. This does not exclude the possibility that DAF-16 activates *pha-4 *transcription, but it suggests there may be multiple transcription factors that regulate *pha-4 *expression.

We employed dauer recovery assays to characterize the genetic interaction between *daf-16 *and *pha-4*. The *daf-16 *mutation and *pha-4 *RNAi have additive effects on inhibition of dauer recovery. A *daf-16; daf-7 *dauer-constitutive mutant treated with *pha-4 *RNAi showed more severe dauer recovery defects compared to either the *daf-16; daf-7 *mutant treated with the control RNAi or the *daf-7 *mutant treated with *pha-4 *RNAi. Therefore, DAF-16 and PHA-4 function in parallel pathways to regulate dauer recovery.

### Other potential regulators of PHA-4

The regulation of *pha-4 *expression and activity is complex. *pha-4 *expression in the intestine is inhibited by the *let-7 *micro RNA, which is essential for the transition from the L4 larval stage to adult [44]. Interestingly, *let-7 *also inhibits DAF-12 expression in hypodermal seam cells [44]. Since micro RNAs usually regulate translation of the mRNA, increased mRNA levels do not guarantee elevated activity. It will be interesting to examine whether PHA-4 protein levels and activities are increased in dauer larvae.

There is a tract of 29 C residues in the second intron of *pha-4 *(Fig. 5A). Deletions in this sequence can be frequently detected in the *dog-1 *(DEAH helicase) mutant [45], which exhibits germline and somatic deletions in genes containing such poly G/C tracts. However, it is not yet known whether this poly C tract plays a regulatory role in *pha-4 *expression. Recently, Updike and Mango [46] have reported a screen for suppressors of *pha-4 *loss-of-function. One of the suppressors (*px63*) turned out to be an allele of *pha-4*, with elevated expression. The *pha-4(px63) *allele carries a 156 bp deletion in the second intron, and an insertion of ≥ 5.3 kb between the 3' end of intron 2 and the middle of exon 5 [46]. Interestingly the deletion overlaps with the polyC tract, and the insertion is likely to overlap with the potential DAF-16 binding site and flanking sequences. Updike and Mango proposed that the DNA sequence rearrangement in *pha-4(px63) *either disrupts a negative regulatory element or introduces a positive element for *pha-4 *expression [46]. It will be interesting to test which mutation is responsible for the elevated *pha-4 *expression and whether the polyC tract or the potential DAF-16 binding site is involved.

### PHA-4 is required for vulval transcription of *lag-2*

*lag-2 *encodes the ligand for Notch signaling pathways in the gonad and vulva. Our results showed that PHA-4 is important for *lag-2 *transcription in vulval cells, but does not affect *lag-2 *expression in distal tip cells for germline development. Vulval transcription of *lag-2 *is regulated by the inductive EGFR/MAPK signaling pathway, and LIN-31 is the transcription factor functioning downstream of this pathway in vulva development [37]. Although we found that PHA-4 binds an element in the *lag-2 *promoter *in vitro*, we did not detect any PHA-4 expression in vulval cells where *lag-2 *is expressed using the partial PHA-4::GFP translational fusion reporter. More work will be required to determine whether PHA-4 directly promotes *lag-2 *transcription, or whether it functions through components of the EGFR/MAPK pathway.

## Conclusion

DAF-16 and PHA-4 play essential roles during dauer recovery, and PHA-4 functions upstream of LIN-12/Notch signaling during vulval development. The dauer/non-dauer decision is determined by food, population density and temperature [5]. The essential functions of PHA-4 in both pharyngeal and reproductive development in response to environmental factors suggest a link between nutrient in-take and reproduction. Hence, the analysis of PHA-4 functions in dauer larvae will help explain how the overall network of transcriptional control is formed, and how different aspects of *C. elegans *development are integrated.

## Methods

### Strains

Strains were maintained as described by Brenner [47]. Strain names and genotypes of animals used were: GR1329 *daf-16(mgDf47)I*, DR2279 *age-1(m875)II*, CB1370 *daf-2(e1370)III*, CB1372 *daf-7(e1372)III*, DR40 *daf-1(m40)IV*, DR2427 *daf-16(mgDf47)I; daf-7(e1372)III*, DR2429 *daf-2(e1370)III; qIs56*(*unc-119; lag-2::gfp)(IV or V)*, DR2454 *daf-2(e1370III) mEx167 [rol-6(su1006) pha-4pE3::gfp]*, DR2455 *daf-2(e1370III) mEx168 [rol-6(su1006) pha-4pE3::gfp]*, DR2458 *daf-2(e1370III) mEx169 [rol-6(su1006) pha-4::gfp]*, DR2459 *daf-2(e1370III) mEx170 [rol-6(su1006) pha-4::gfp]*, DR2460 *daf-2(e1370III) mEx171 [rol-6(su1006) pha-4::gfp(M)]*, and DR2461 *daf-2(e1370III) mEx172 [rol-6(su1006) pha-4::gfp(M)]*.

### RNAi

A 1.3-kb coding region of the *pha-4 *gene was amplified from *C. elegans *genomic DNA using primers 5' CGG AAT TCG TTT TAC CAC TGG CAC CAC 3' and 5' CCC AAG CTT CTG GTA TAC TCC GTT GGT G 3'. The PCR products were cloned into the RNAi vector L4440 between the *Eco*R I and *Hind *III sites. The *pha-4 *RNAi construct was transformed into *E. coli *HT115(DE3). RNAi bacteria cultivation and double-stranded RNA induction were performed as described by Kamath *et al*. [32]. In all RNAi assays, *E. coli *HT115(DE3) carrying the empty RNAi vector L4440 was fed to the same strain as controls.

### Dauer recovery assays

About ten Daf-c gravid adults were transferred to RNAi plates, and allowed to lay eggs for 4 to 6 hours before they were removed. The plates were incubated at 25°C for three days until dauer larvae formed constitutively. The dauer larvae were then transferred to fresh RNAi plates and incubated at 15°C to resume development. Animals that became L4 larvae or young adults after 3 days at 15°C were scored as recovered. Gonad and vulva morphologies of these adult animals were examined using a Zeiss Axioscope with Nomarski optics.

### RT-PCR

Total RNA was extracted using the Trizol reagent (Invitrogen) following the manufacturer's instructions. Reverse transcription followed by PCR reactions were performed to determine gene transcription levels from different samples as previously described [48]. The following gene-specific primers were used to amplify transcripts of interest: *pha-4*, 5' GCG GAG CTC ATG AAC GCT CAG GAC TAT CTG 3' and 5' CGC AAG CTT TAG GTT GGC GGC CGA GTT C 3' ; *rpl-21*, 5' ATG ACT AAC TCC AAG GGT C 3' and 5' TCA CGC AAC AAT CTC GAA AC 3'

### *pha-4::gfp *reporters

A 5.5-kb DNA fragment that contains the 1.5-kb promoter through part of the third exon of *pha-4 *was amplified from *C. elegans *genomic DNA using primers 5' CAA CGA GAG GGC ATG CTG TGA AC 3' and 5' CGG GAT CCT GAT ATG GTT GGT AGT TTA ACG 3'. The PCR products were cloned into the vector pPD95.67 (a gift from Dr. Andrew Fire) between the *Sph *I and *Bam*H I sites. The *pha-4::gfp *clone (50 ng/μl) was injected into the ovaries of *daf-2(e1370) *young adults along with the pRF4 *rol-6(su1006) *dominant transformation marker (100 ng/μl).

To test whether the potential DAF-16 binding site in the second intron of *pha-4 *is functional *in vivo*, a 3.8-kb DNA fragment was amplified from *C. elegans *genomic DNA using primers 5' ACA TGC ATG CGT AAG GCA CCA GTT ATT TTC TG 3' and 5' CGG GAT CCG GCC TGC AAG AAA AAA ATT GAA AG 3'. The PCR products were cloned into the vector pPD95.67 between the *Sph *I and *Bam*H I sites. The potential DAF-16 binding site (TATTTAC) was mutated to GCGGGCA using the QuickChange site-directed mutagenesis kit (Stratagene). The constructs (50 ng/μl) with normal or mutant binding sites were co-injected into the ovaries of *daf-2(e1370) *young adults along with the pRF4 (100 ng/μl) marker.

#### Note

It has been reported that PHA-4 function is required for exit from the dauer stage and for development of the vulva  [28,46].

## Abbreviations

SAGE, serial analysis of gene expression; RT-PCR, reverse transcriptase polymerase chain reaction; Daf, dauer formation; RNAi, double stranded RNAi-mediated interference; GFP, green fluorescent protein; DTC, distal tip cell.

## Authors' contributions

DC and DLR. designed the experiments and drafted the manuscript. DC carried out the experiments.
